# WildSilkbase: An EST database of wild silkmoths

**DOI:** 10.1186/1471-2164-9-338

**Published:** 2008-07-17

**Authors:** KP Arunkumar, Archana Tomar, Takaaki Daimon, Toru Shimada, J Nagaraju

**Affiliations:** 1 Centre of Excellence for Genetics and Genomics of Silkmoths, Laboratory of Molecular Genetics, Centre for DNA Fingerprinting and Diagnostics, ECIL road, Nacharam, Hyderabad – 500 076, India; 2 Department of Agricultural and Environmental Biology, The University of Tokyo, Yayoi 1-1-1, Bunkyo-ku, Tokyo 113, Japan

## Abstract

**Background:**

Functional genomics has particular promise in silkworm biology for identifying genes involved in a variety of biological functions that include: synthesis and secretion of silk, sex determination pathways, insect-pathogen interactions, chorionogenesis, molecular clocks. Wild silkmoths have hardly been the subject of detailed scientific investigations, owing largely to non-availability of molecular and genetic data on these species. As a first step, in the present study we generated large scale expressed sequence tags (EST) in three economically important species of wild silkmoths. In order to make these resources available for the use of global scientific community, an EST database called 'WildSilkbase' was developed.

**Description:**

WildSilkbase is a catalogue of ESTs generated from several tissues at different developmental stages of 3 economically important saturniid silkmoths, an Indian golden silkmoth, *Antheraea assama*, an Indian tropical tasar silkmoth, *A. mylitta *and eri silkmoth, *Samia cynthia ricini*. Currently the database is provided with 57,113 ESTs which are clustered and assembled into 4,019 contigs and 10,019 singletons. Data can be browsed and downloaded using a standard web browser. Users can search the database either by BLAST query, keywords or Gene Ontology query. There are options to carry out searches for species, tissue and developmental stage specific ESTs in BLAST page. Other features of the WildSilkbase include cSNP discovery, GO viewer, homologue finder, SSR finder and links to all other related databases. The WildSilkbase is freely available from .

**Conclusion:**

A total of 14,038 putative unigenes was identified in 3 species of wild silkmoths. These genes provide important resources to gain insight into the functional and evolutionary study of wild silkmoths. We believe that WildSilkbase will be extremely useful for all those researchers working in the areas of comparative genomics, functional genomics and molecular evolution in general, and gene discovery, gene organization, transposable elements and genome variability of insect species in particular.

## Background

Sudden spurt in sequencing projects in recent years has resulted in exponential increase in the genome sequence repertoire of species that are close relatives of many model organisms. Availability of the sequence resources has accelerated comparative genomic analysis and has thus added to our understanding of organismal biology of these species. In fact new insights into human genome have come only after the sequences of related species such as chimpanzee, monkey and ape were published. However, in the insect order Lepidoptera, which consists of many economically important insects such as silkmoths, agriculture pests and beautiful butterflies, only the domesticated silkworm, *Bombyx mori *has achieved the distinction of the most well studied insect next only to Drosophila. Therefore, comparative genomics in this order is still in its infancy.

Functional genomics has particular promise in silkworm biology for identifying genes involved in synthesis and secretion of silk, pathways involved in processes such as insect-pathogen interactions and sex determination mechanisms. Among lepidopterans, several databases such as Silkbase [1] for ESTs, Kaikobase [2] for whole genome sequence, Silkdb [3] for ESTs and whole genome sequence, and Silksatdb [4] for microsatellites, have been developed for *B. mori*. Apart from *B. mori*, more than 13,000 ESTs have been made available for butterflies through Butterflybase [5] and 32,217 ESTs for the pest species, *Spodoptera frugiperda *through Spodobase [6]. However, wild silkmoths are least represented owing largely to non-availability of genomic resources in these species. Hence, generation and utilization of genomic information from wild lepidopterans will be extremely useful in understanding these species at molecular level.

The members of family Saturniidae, collectively known as saturniids, are among the largest and most spectacular of the Lepidoptera, with an estimated 1,300 to 1,500 different described species distributed worldwide [7]. The Saturniidae family includes the giant silkmoths, royal moths and emperor moths. The muga silkmoth, *Antheraea assama *(n = 15), confined to the North-eastern states of India, is the least understood and unique species among saturniid moths. The silk proteins of this species have not been studied so far despite their unique properties of providing golden lustre to the silk thread. *Samia cynthia ricini *(n = 13) a multivoltine silkworm commonly called as 'eri silkworm' is known for its white or brick-red eri silk. It is distributed in India, China and Japan. Its ecoraces (~16) are distributed across the Palaearctic and Indo-Australian biogeographic regions. The tropical Indian tasar silkmoth, *Antheraea mylitta *(n = 31) is a natural fauna of tropical India, represented by more than 20 well-described, genetically distinct ecoraces. Pursuing genetics and genomics of saturniids will be of significance for the following reasons: a) Typical of lepidopterans, *B. mori *females are heterogametic, with a ZW chromosome constitution; males are ZZ. Sex chromosomes are considered to be under evolutionary constraints different from those of autosomes. W chromosome is reported to be strongly female determining [8]. The sex chromosome system of saturniid silkmoth *A. assama*, on the other hand, is ZZ/ZO as compared to ZZ/ZW observed in other silkmoths. Comparative study of the sex determining genes, would thus reveal diverse sex determination mechanisms in silkmoths, b) Photoperiod plays an important role in the life history traits of wild silkmoths and hence it is important to investigate the genes involved in circadian rhythm, c) Silk fibres of different wild silkmoths show vast differences in their tenacity, texture, lustre and many other biophysical properties. In the light of these, it is interesting to study the genes encoding the silk proteins of wild silkmoths and compare them with those of mulberry silkmoth, and d) Information on immune response genes in these species can throw light on diversity of immune repertoir in these moths and may lead to identification of novel immune genes.

The rapid and ever increasing deposition of ESTs of various organisms in dbEST database of NCBI shows the role of EST projects in advancing genomic science in eukaryotes. Considering the potential scientific benefits, in the current study we generated 57,113 ESTs in three wild silkmoth species, *A. assama*, *S. c. ricini *and *A. mylitta *from different tissues at various developmental stages (Table 1). These ESTs and resulted unigenes were annotated for Gene Ontology (GO) terms, simple sequence repeats (SSRs) and single nucleotide polymorphisms (SNPs). To get insight into conservation and divergence of genes among silkmoths and model insects, we compared the putative unigenes of the three wild silkmoth species among themselves and with those of four insect species, *B. mori*, *Drosophila melanogaster*, *Apis mellifera *and *Tribolium castaneum*. In order to make the EST resources of wild silkmoths and their annotations available for the use of scientific community an EST database called 'WildSilkbase' was developed.

**Table 1:** Details of cDNA libraries, number of ESTs generated in each wild silkmoth species and results of EST analysis.

		**Total no. of ESTs**	**No. of contigs**	**No. of singletons**	**No. of unigenes**
***Antheraea assama***	**10 libraries**	**35,722**	**2,260**	**5,937**	**8,197**

***Tissue type***	***Developmental stage***				

Embryo	96 hours after oviposition	11,502			
Brain	Fifth instar	5,299			
Testis	Fifth instar	4,235			
Ovary	Fifth instar	3,891			
Fatbody	Fifth instar	2,318			
Midgut	Fifth instar	2,439			
Posterior silkgland	Fifth instar	2,543			
Middle silkgland	Fifth instar	1,031			
Epidermis	Fifth instar	1,386			
Compound eyes	Fifth instar	1,078			

***Samia cynthia ricini***	**3 libraries**	**19,979**	**1,593**	**3,528**	**5,121**

***Tissue type***	***Developmental stage***				

Embryo	96 hours after oviposition	6,647			
Fatbody I	12–24 hours after injection of *E. coli *into hemocoel of fifth instar larvae	6,681			
Fatbody II	12–24 hours after injection of *Candida albicans *into hemocoel of fifth instar larvae	6,651			

***Antheraea mylitta***	**1 library**	**1,412**	**166**	**554**	**720**

***Tissue type***	***Developmental stage***				

Fatbody	24 hours after injection of *E. coli *into hemocoel of fifth instar larvae	1,412			

**Total**	**14 libraries**	**57,113**	**4,019**	**10,019**	**14,038**

## Construction and content

EST sequences were generated from 3 wild silkmoth species by sequencing the cDNA clones amplified from mRNA isolated from several tissues at different developmental stages. All the protocols employed for RNA extraction, cDNA library preparation and EST processing pipeline are available online in the database and can be accessed from 'Protocols' section. The ESTs were further processed with Phred program [9] for base calling DNA sequence traces. A cut off Phred score of 15 was assigned to extract quality sequences from chromatograms. In order to enhance the quality of sequences, ESTs were screened for presence of vector sequences and subsequently detected vector sequences were then removed using 'Cross Match' program. EST reads with length less than 100 bp were discarded. Majority of ESTs included in the database are having the lengths ranging between 400–600 bp.

To produce non redundant EST dataset for further functional annotation and comparative analysis, 57,113 ESTs were clustered and assembled through TGICL package [10] with the CAP3 [11] default options. Based on regions of similarity, EST sequences were merged into contigs. A total of 14,038 EST clusters consisting of 4,019 contigs and 10,019 singletons, putatively regarded as unigenes, was generated (Table 1). From these unigene sequences, poly-A tails were trimmed using TrimEST program of EMBOSS [12]. Trimmed unigene sequences thus obtained were annotated for GO [13]. The GO annotation is based on the closest homologues identified by BLAST search against Seqdblite FASTA sequence flat file [14]. All the unigenes were assigned a biological process, molecular function and cellular component using GO database. ESTs are potential resources for SSR and SNP marker discovery and hence they were screened for SSRs by using Tandem Repeats Finder (TRF) [15]. For extraction of repeats, we assigned the following TRF parameters: match = 2, mismatch = 3, indel = 5, match probability = 0.8, indel probability = 0.1, minimum score = 25 and maximum period = 10. *A. mylitta *EST sequence dataset was further analysed for potential SNPs in cDNA sequences (cSNPs) using SEAN SNP Prediction Program with default settings [16]. A total of 118 cSNPs was predicted in 1412 EST sequences. These predicted cSNPs, after experimental validation, will be useful for the analysis of genetic variation and population structure of *A. mylitta *populations.

## Utility and discussion

**Figure 1:**
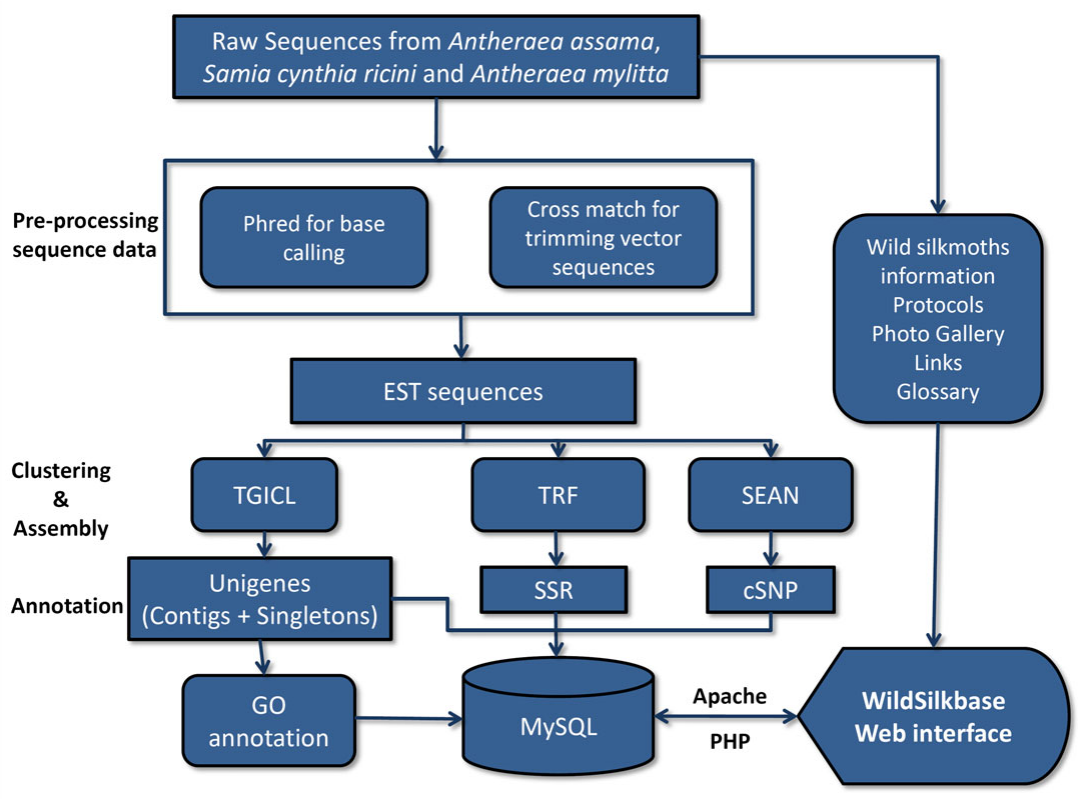
WildSilkbase organisation and implementation.

WildSilkbase is designed to provide a platform for storage of sequence data and access to annotated information on ESTs of wild silkmoths. The database has been developed using MySQL relational database system, and its web interface has been generated using PHP scripts. It operates under an Apache web server on a Fedora Linux system. The data model for raw and processed data of WildSilkbase is shown in Figure 1.

**Figure 2:**
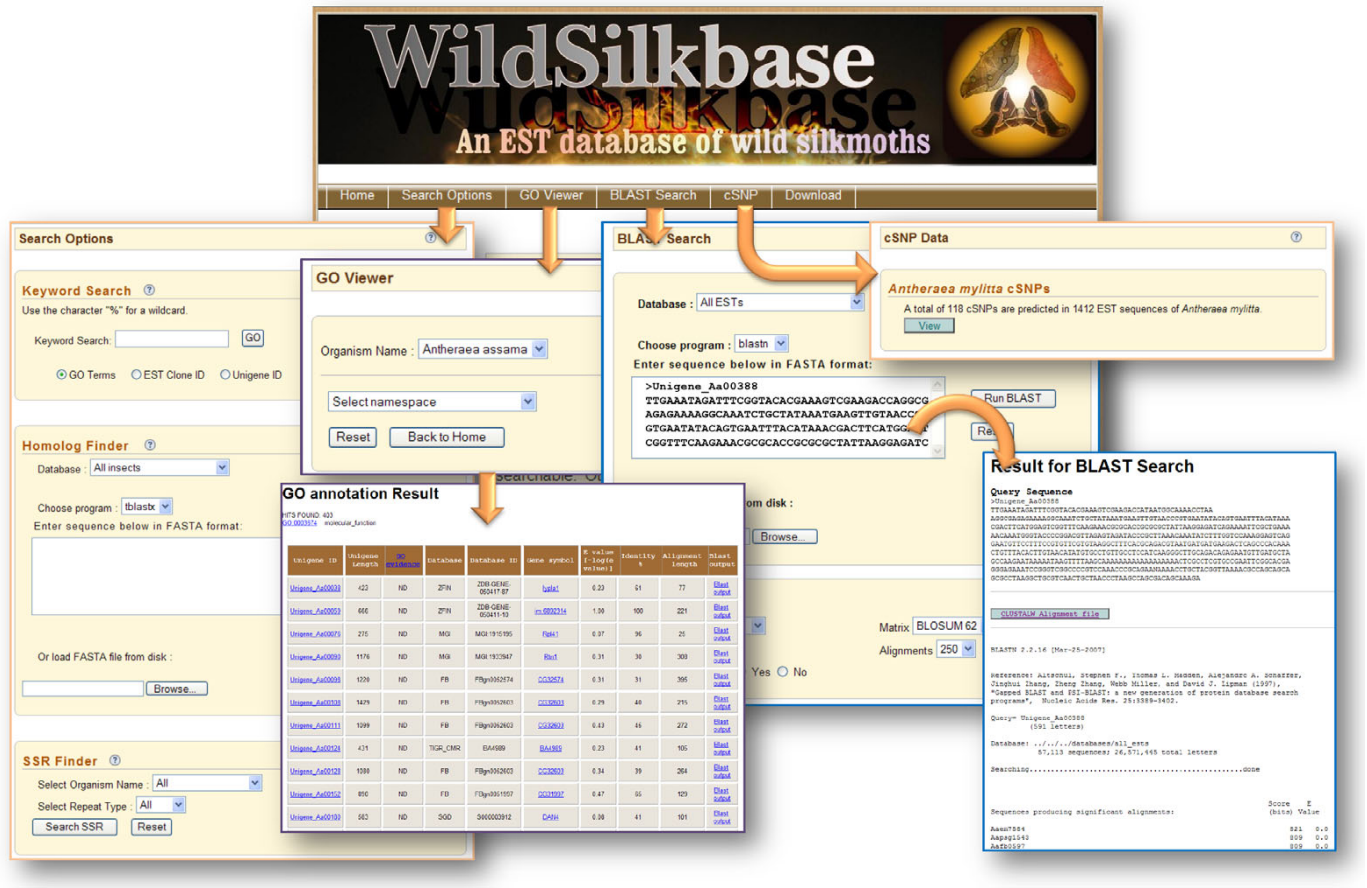
Web-shots of the database showing WildSilkbase query options, keyword search, homologue finder, SSR finder, GO viewer, BLAST search, cSNP data and download option.

Database can be inspected via four different HTML pages to allow distinctive queries. The 'Search Options' page provides three different options namely, Keyword Search, Homolog Finder and SSR Finder, for searching the database. The 'Keyword Search' gives access to search the database by GO terms, EST clone ID and Unigene ID. 'Homolog Finder' allows end user to search for the homologue of the query sequence against 6 different insect species (*Aedes aegypti*, *Anopheles gambiae*, *A. mellifera*, *B. mori*, *D. melanogaster *and *T. castaneum*) based on BLAST search. 'SSR Finder' provides access to data on SSRs of wild silkmoth ESTs included in the database. Search results can be limited by the selection of SSRs of specific type belonging to a particular species. The output generated is displayed in a tabulated format (Figure 2).

To categorise transcripts by function, we utilized the GO classification. The 'GO Viewer' interface is designed to browse GO terminologies as a tree of terms. The number next to GO term represents the number of gene products annotated to that term which are included in the database and selected in the current view. BLAST [17] search offered by WildSilkbase allows users to compare any query sequence against *A. assama*, *S. c. ricini *and *A. mylitta *ESTs and putative unigene sequence datasets. BLAST search results are returned directly to the user's web browser in HTML format (Figure 2). The sequence IDs on the BLAST result page are further linked to respective sequence information such as organism name, tissue of origin, sequence length, unigene ID and sequence. A link to ClustalW [18] alignment file of the sequences matched in the databases is also provided on the result page.

The 'cSNP' web page provides direct access to cSNPs of *A. mylitta*. The results include information such as, contig ID, contig sequence length, the ESTs included in the contig, SNP location, alleles and consensus sequence.

The database also provides information on wild silkmoth biology, cDNA library construction and EST processing pipeline. The 'Picture Gallery' section has been incorporated in the database to give access to pictures of wild silkmoths. Links to several other databases and resources related to ESTs and insects are provided on 'Useful Links' webpage. A 'General Help' page is included for easy and efficient use of the database. The technical terms occurring in the database are hyperlinked to the 'Glossary' page for quick reference. In general, WildSilkbase allows the users to access all applications. The EST sequences of wild silkmoths are also deposited in NCBI and can be accessed at the NCBI EST sequence database, dbEST (accession numbers: *A. assama*; FE952359-FE963860 and FG203277-FG226965, *A. mylitta*; EB742119-EB743530, *S. c. ricini*; DC858270-DC878540).

### Data analysis

#### Gene Ontology annotation

**Figure 3:**
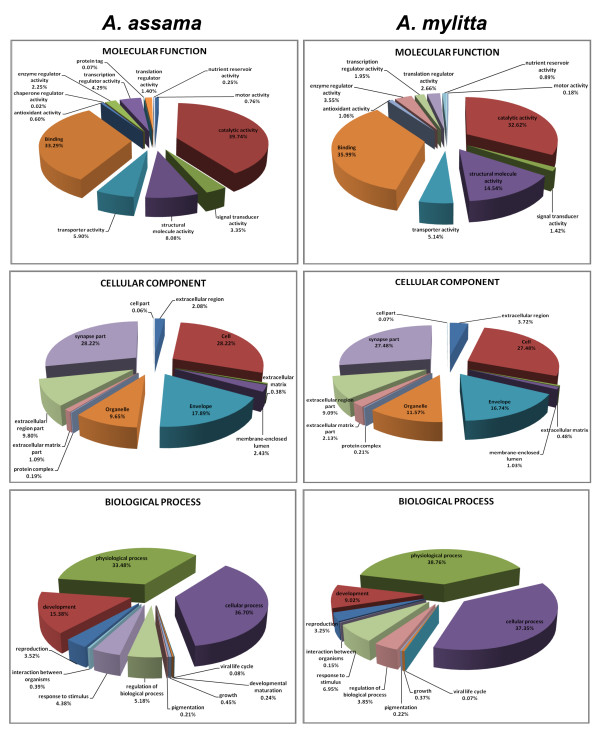
**Gene Ontology representation of *A. assama *and *A. mylitta *clusters is shown for each organizing principle of GO: biological process, cellular component and molecular function**. The chart is based on percentage representation of GO mappings.

**Figure 4:**
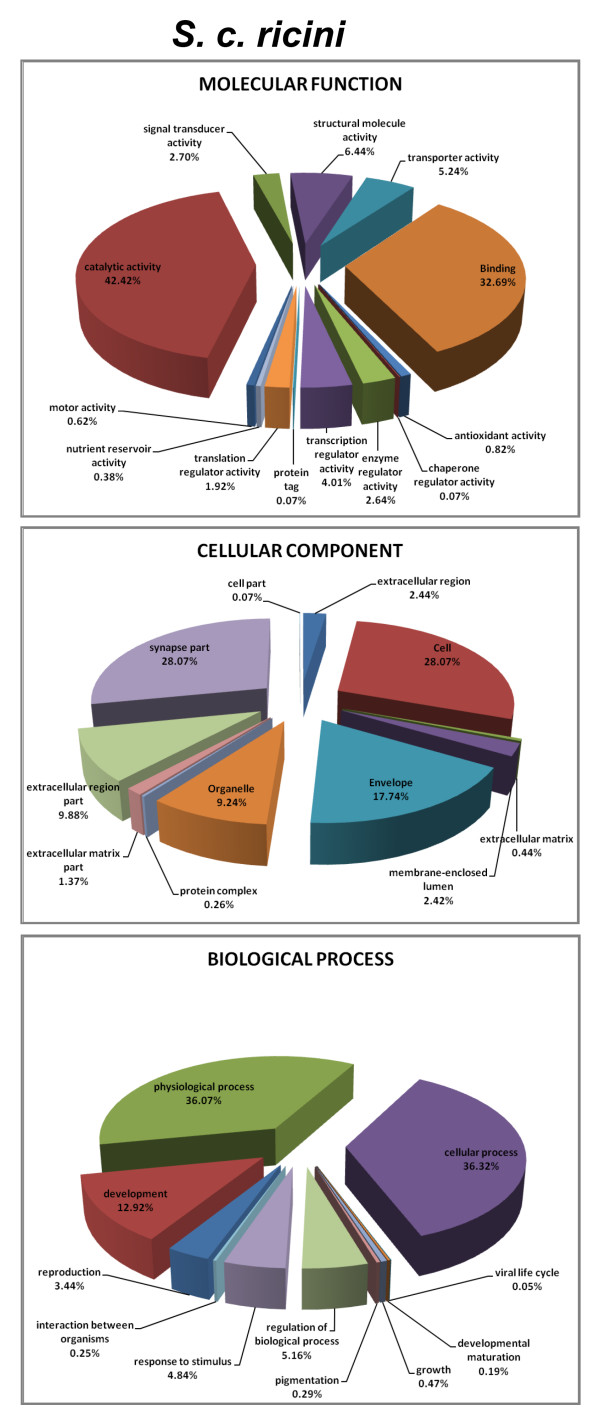
Percentage representation of gene ontology (GO) mappings for *S. c. ricini *clusters by biological process, cellular component, and molecular function.

GO annotation generates a dynamic controlled vocabulary that can be applied to all organisms, even while knowledge on genes and proteins in cells is still accumulating. The closest annotated homologue in the GO database was used for assigning these categories. Based on the GO annotation of the closely related homologues, the ESTs were assigned a molecular function, biological process and cellular component from the GO database [19]. To accomplish this, the Seqdblite FASTA sequence flat file was downloaded from the GO database. By running BLAST against Seqdblite, the closest homologue was identified. From the BLAST output, molecular functions, biological process and cellular localization were parsed by building an in-house GO database in MySQL from the GO-term database flat file, downloaded from GO Database Downloads [20]. The output is graphically represented (Figures 3 and 4).

GO mapping to molecular function revealed that a majority of genes from the tissue transcriptomes of wild silkmoths have almost equal distribution for 'binding' function (property of binding macro-molecules) and catalytic activity. The next abundant molecular function observed in the transcriptomes was structural molecule activity. In case of biological process, majority of the ESTs belonged to the category of physiological processes and cellular processes. Based on cellular localization results, most of the gene products were found to be localized in cell and synapse part (Figures 3 and 4). Putative unigenes for each category of GO, of any of the three wild silkmoth species can be browsed, viewed and downloaded from the 'GO Viewer' option of WildSilkbase.

#### Comparative genomics

Information on sequence similarity among genomes is a major resource for finding functional regions and for predicting their functions. Comparison of the genomes of closely related species is useful for finding the key sequence differences that may account for the differences in the organisms. Comparative genomics is thus a powerful and burgeoning discipline and has become more and more informative as genomic sequence data accumulate [21]. The lepidopteran insects have taxonomically specific biological phenomena including sex-determination, pheromone-dependent sexual communication, silk production, silk protein organisation, circadian rhythms, insect-plant interactions and insect-microbe interactions. Comparing genes of the wild silkmoths with other lepidopterans and other model insect species would shed light on conservation and divergence of different gene families. In the present study we compared the putative unigenes of the three wild silkmoth species between each other and with the unigenes of four insect species, *B. mori*, *D. melanogaster*, *A. mellifera *and *T. castaneum*.

**Figure 5:**
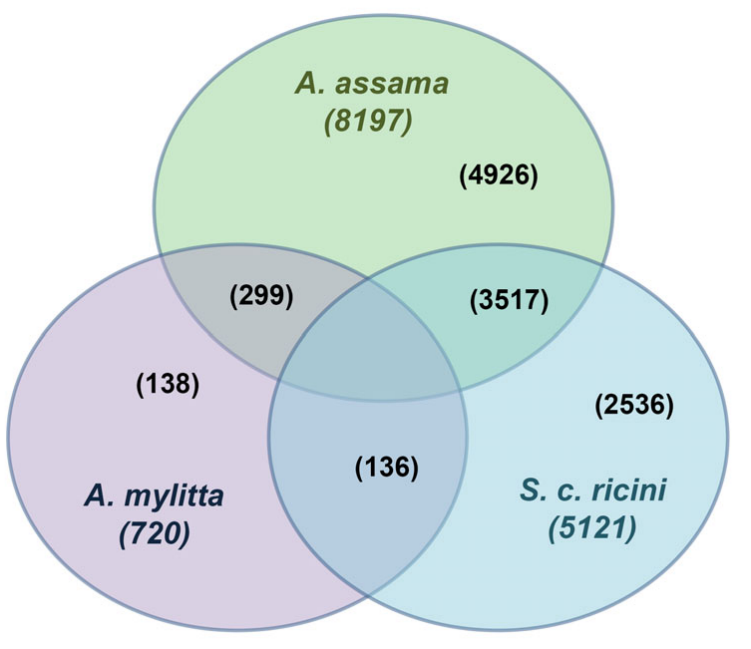
Venn diagram illustrating the number of unigenes shared among the 3 wild silkmoths, *A. assama*, *S. c. ricini *and *A. mylitta*.

The putative unigenes of each of the three wild silkmoth species were compared among each other using TBLASTX at the cutoff E-value of 1e-5. The resultant homologs between each pair and homologs common to all the three species and unique genes present only in one of the species are represented graphically (Figure 5). Gene comparisons, for example, revealed that *A. mylitta *has more number of homologous genes with *A. assama *than with *S. c. ricini*. This is in accordance with the phylogenetic relationship among silkmoth species [22], as both of them belong to the genera *Antheraea*.

**Figure 6:**
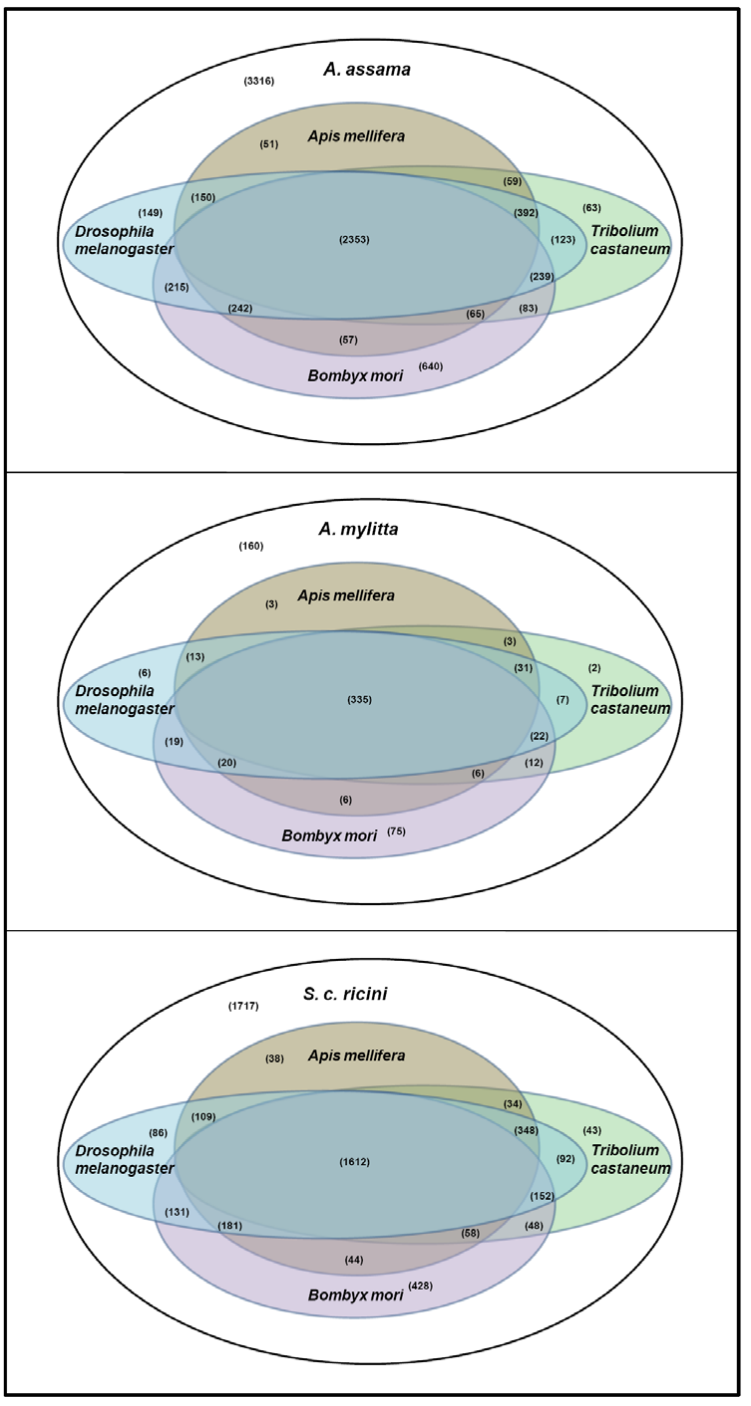
Venn diagrams showing the number of shared and species-specific genes among each of the 3 wild silkmoth species and 4 model insect species.

A total of 9939, 17178, 9747 and 9028 unigene sequences of *B. mori*, *D. melanogaster*, *A. mellifera *and *T. castaneum *respectively, was downloaded from Unigene database of NCBI [23]. Putative unigenes from each of the three wild silkmoth species were compared with these four insect species through TBLASTX search with E-value limit of 1e-5. Total number of homologs obtained was plotted on the graph. In all these comparisons *B. mori *was found to share the highest number of homologous genes with all the three wild silkmoth species and *D. melanogaster *appearing next only to it, followed by *T. castaneum *and *A. mellifera *(Figure 6), which is expected as all the wild silkmoth species and *B. mori *share common ancestor [24] and belong to the order, Lepidoptera.

## Conclusion

WildSilkbase aims to provide user-friendly access to EST data on wild silkmoths. Database will be continuously updated as and when the new information is available on wild silkmoth ESTs. We welcome feedback from users for further improvement of this database. Researchers working on silkmoths are encouraged to submit their wild silkmoth EST data to WildSilkbase, so that it can be made a single window portal for all information on wild silkmoths. In future, additional features will be added. Enhanced functional annotation would be mined from other cluster and pathway databases to make WildSilkbase more user-friendly and to extract maximum information from this database. WildSilkbase, we firmly believe, will be extremely useful for the researchers working in the areas of ecology, evolution, functional and comparative genomics, genetics and biochemistry of insects.

## Availability and requirements

WildSilkbase is freely available through . All questions, comments and suggestions should be sent to jnagaraju@cdfd.org.in.

## Authors' contributions

JN conceived and designed the project. KPA and JN constructed cDNA libraries from *A. assama *tissues and sequenced ESTs. TD and TS constructed cDNA libraries from *S. c. ricini *tissues and sequenced ESTs. KPA and AT carried out the data analysis and database construction. KPA, AT and JN wrote the manuscript. All the authors read and approved the final manuscript.
